# Advanced non-small cell lung cancer: on relapse rechallenge the tumor, not the patient

**DOI:** 10.1186/1756-0500-3-195

**Published:** 2010-07-14

**Authors:** Marios E Froudarakis, Evangelos Briasoulis

**Affiliations:** 1 Department of Pneumonology, Medical School of Alexandroupolis, Democritus University of Thrace, Greece; 2 Department of Medical Oncology, Medical School, University of Ioannina, Greece

## 

Non-small cell lung cancer (NSCLC) is the leading cause of cancer-related mortality in the world. The majority of NSCLC patients are diagnosed with advanced-stage disease and almost all except a tiny minority will develop incurable metastatic disease. The standard of care for such patients with a good performance status (PS) is currently platinum based chemotherapy, which can offer palliation of symptoms but only modest survival improvement [1-3]. Nearly all patients who receive systemic therapy for advanced-metastatic NSCLC will eventually relapse [4]. This is an unquestionable reality. Physicians who deal with medical management of NSCLC patients who failed first line therapy are often puzzled with three key questions: if, what, and whom, to offer a second line therapy. Existing evidence is unfortunately weak, if not confusing, to address such issues. It is therefore apparent that for optimal medical management of these patients we need molecular and/or clinical biomarkers to safe-guide therapeutic decisions.

Randomized clinical trials have shown that second-line chemotherapy in NSCLC patients who failed or relapsed first-line therapy, can offer modest and marginal survival benefits at the cost of considerable toxicity. Yet patients enrolled in such studies were not quite representative of this group of patients [5]. Treatment options available for previously treated advanced-metastatic NSCLC are single agent docetaxel, pemetrexed, and epidermal growth factor receptor inhibitors (erlotinib and gefitinib) [6]. It should be stressed herein that only single-agent therapies have been approved and are recommended for clinical use in this clinical setting. Combination chemotherapy failed to show survival benefits when compared with single agent treatment in the clinical setting of second line chemotherapy but increased toxicity [7]. Results were also disappointing when chemotherapy was combined with anti-VEGF antibody bevacizumab [8].

Approximately 30% of patients with advanced-metastatic NSCLC will also receive second-line chemotherapy [9]. It is therefore obvious that we need biomarkers to help us select patients who have chances to get benefit from therapy and pick the best therapeutic option for each individual. Several investigators have tried to profile NSCLC patients who can tolerate second line chemotherapy [10,11]. Treatment tolerability is always a significant parameter for patients who are offered palliative therapy. In advanced NSCLC, patients with a poor performance status (PS 2 and worse) have shown to fare poorly to platinum-based doublets even when treatment is given upfront [12]. Although newer agents have been tested in doublets or in combination with novel platinum compounds [13], no benefit was observed when compared to single agent chemotherapy [7]. Currently it is recommended that such patients should be offered single-agent chemotherapy to low the risk of unwanted toxicity, which is expected high in such cases [6,7]. Moreover, there is no evidence to support second-line treatment of patients with poor performance status [14].

A recent analysis, which included data from 1239 patients, demonstrated that prognosis is significantly influenced by gender (worse in males), PS, tumor histology (worse in squamous and other histology versus adenocarcinoma), stage (worse in IV versus IIIB), type of previous treatment (worse for patients pretreated with platinum) and response to first-line (worse for patients not obtaining objective response). The authors devised a summary index (score) that could discriminate patients into three categories according to probability of survival [11]. However, prospective studies aiming to identify patients with relapsed advanced-metastatic NSCLC who might be benefited of second-line chemotherapy are surprisingly missing. The paper of Belbaraka et al. [15], from Centre Léon Bérard, Lyon, France, stresses this issue in a prospective way. The authors identified poor PS as the primary indicator for failure to second line chemotherapy. Despite its limitations, (i.e. small number of patients, heterogeneity of chemotherapy administered and lack of quality-of-life data), this prospective study showed that poor PS should make treating physicians reluctant in advising second-line chemotherapy for patients with relapsed NSCLC [15].

Nevertheless it should be noticed that chemotherapy is not the only systemic therapy available for NSCLC patients today. Small molecule inhibitors of cancer-associated disordered epidermal growth factor receptor (EGFR) are now approved for NSCLC patients with EGFR mutated tumors [16]. We have previously proclaimed that personalized therapeutic management based on molecular predictors should be considered the most proper approach in terms of good clinical practice, in the era of cancer targeted therapy [17]. Interestingly, it has been shown that such therapies can substantially benefit patients if only offered to molecularly defined target populations irrespectively of previous therapy. A recent study showed that gefitinib is equally effective in patients affected by EGFR mutated tumors in chemo-naive patients and in chemotherapy-treated patients [18-20].

Whether we should treat patients who relapse after second line chemotherapy is another issue [21]. Massarelli et al. conducted a retrospective analysis of over 700 patients who had received two or more prior chemotherapy regimens for recurrent-metastatic NSCLC. They found that median survival and response rates decreased steadily with each subsequent regimen. The objective responses became infrequent and of short duration and toxicities increased considerable [22]. Understandably, the vast majority of patients who receive second-line therapy are not expected to qualify for a third-line treatment, because of compromised organ functions, clinical deterioration and development of resistance to chemotherapeutics. Besides, we lack phase III data to support routine use of chemotherapy in the third-line setting [3]. For these patients, supportive care is the only reasonable option, in addition to experimental treatments in clinical trials [3].

Symptom palliation and Quality of Life are primary goals of therapy for patients with advanced-metastatic NSCLC upon tumor relapse. Therefore, «gentle» effective therapies, as are weekly and metronomic regimens should rather be favored in cases of relapsed NSCLC patients who are at risk of severe toxicity [23,24].

Finally, when making treatment decisions for patients with relapsed advanced NSCLC we, physicians, should always be simple and wise in our approach. As a rule of thumb, we should preferably opt a targeted or gentle therapy and should let patient's performance status remind us that we may try rechallenge the tumor but not the patient.

## Competing interests

The authors declare that they have no competing interests.

## Authors' contributions

MEF and EB have contributed equally to this manuscript.
